# Investigating the Performance of Bi-Static GPR Antennas for Near-Surface Object Detection

**DOI:** 10.3390/s19010170

**Published:** 2019-01-05

**Authors:** Xianyang Gao, Frank J. W. Podd, Wouter van Verre, David J. Daniels, Anthony J. Peyton

**Affiliations:** School of Electrical and Electronic Engineering, The University of Manchester, Manchester M13 9PL, UK; frank.podd@manchester.ac.uk (F.J.W.P.); wouter.vanverre@manchester.ac.uk (W.v.V.); david.daniels@manchester.ac.uk (D.J.D.); a.peyton@manchester.ac.uk (A.J.P.)

**Keywords:** ground penetrating radar, antenna, optimisation, geometrical configuration, bowtie, bi-static

## Abstract

Antennas are an important component in ground penetrating radar (GPR) systems. Although there has been much research reported on the design of individual antennas, there is less research reported on the design of the geometry of bi-static antennas. This paper considers the effects of key parameters in the setup of a GPR head consisting of a bi-static bow-tie pair to show the effect of these parameters on the GPR performance. The parameters investigated are the antenna separation, antenna height above the soil, and antenna input impedance. The investigation of the parameters was performed by simulation and measurements. It was found when the bi-static antennas were separated by 7 cm to 9 cm and were operated close to the soil (2 cm to 4 cm), the reflected signal from a near-surface object is relatively unaffected by height variation and object depth. An antenna input impedance of 250 Ω was chosen to feed the antennas to reduce the late-time ringing. Using these results, a new GPR system was designed and then evaluated at a test site near Benkovac, Croatia.

## 1. Introduction

Ground penetrating radar (GPR) has many uses and has been employed as a tool for many decades. GPR radiates electromagnetic waves into the ground and detects the discontinuity of the di-electric properties of objects under the soil [1].

This paper focusses on the detection of near-surface objects. In this application, the electro-magnetic signal generally has a centre frequency in the low GHz region, and it is classed as ultra-wideband (UWB).

In many GPR applications, the antennas are held virtually touching (<1 cm) the ground surface, but for some “proximal” applications, the sensor head is held a few centimetres above the soil. A recent application of proximal near surface GPR is for anti-personnel (AP) landmine detection.

Inductive metal detection (MD) has been used to find buried landmines for many decades [2]. However, modern AP landmines have evolved to contain very little metal [3], typically less than a few grams [4], making them harder to find with MD technology and as a result, false alarm rates above 100 per landmine have been reported [5,6] often caused by the unwanted signal from metallic clutter items in the ground. This makes humanitarian de-mining a time-consuming and costly operation, and the cost of clearance is estimated to fall between 300 USD to 1000 USD per land-mine removed [7], compared to a production cost of 3 USD to 30 USD [8].

Proximal near surface GPR can be deployed to, both reduce the false alarm rate from the metal clutter, and to detect the non-metallic content. However, GPR suffers from its own false alarm or clutter sources such as animal burrows, roots, and rocks. Therefore it is usually combined with a MD sensor, where the output from both sensors can be fused to lead to a reduction in the overall false alarm rate [9,10]. Field trials with dual-modality landmine detectors have shown that this approach can lead to reductions in the false alarm rate for humanitarian de-mining between 77% and 96.5% [11,12,13].

Researchers have investigated a wide variety of antenna types for GPR application, such as bowtie [14], Vivaldi [15], spiral [16], and transverse electromagnetic (TEM) horn [17] types. For each antenna investigation, such as bowtie antennas, researchers have used loaded antenna [18,19], designed a specific antenna shape [20,21], or designed a well-performing balun [22,23] to produce a single UWB antenna.

Bowtie antennas can be seen as a planar cut through a bi-conical antenna. Bowtie antennas are smaller and lighter than bi-conical antennas due to their two-dimensional nature, and can be manufactured using traditional printed circuit board (PCB) manufacturing techniques. While their input bandwidth is not as wide as that of bi-conical antennas, they are nonetheless considered wide-band antennas with many applications [24]. Brown and Woodward investigated the radiation characteristics of both bi-conical and bowtie antennas [25], and Carrel described an analytical solution for their input impedances, showing that the input impedance of a bowtie antenna depends on its flare angle [26].

Bowtie antennas are commonly used in GPR applications [1,27], and multiple commercial GPR systems are known to use bowtie antennas [28], which is why this antenna type was chosen for this study.

For the detection of near-surface objects, it is critical to design a well-damped antenna system with a short impulse response exhibiting minimum late-time ringing, to avoid the masking of shallow targets [29]. Internal reflections between the end of the antenna and the feed-point are the main cause for the late-time ringing [29]. Different modifications to the basic bowtie antenna design have been suggested with the aim of optimising the radiated pulse shape. A popular approach is to introduce resistive loading into the antenna structure, as first described by Wu and King [30] for di-pole antennas.

The Wu-King profile of resistive loading defines the profile of impedance per unit length which reduces the reflection from the end of the antenna to zero. However, the steep increase in resistance required near the antenna end can be difficult to achieve when using discrete resistors [31], and can be impractical even when using resistive sheet materials [32]. Resistive loading reduces antenna radiation efficiency [30,31,32,33], and as result, research has been done on reactive loading instead (either inductive or capacitive [34]), as it is non-dissipative. A combination of resistive and capacitive loading can also be used to minimise the loss in radiation efficiency [19]. This paper provides another approach to reducing the late-time ringing of antennas by changing the antenna input impedance.

Generally, antennas are designed in isolation, and there has been little literature on bi-static antenna configurations and their geometry, specifically on the interaction between the antenna separation, the antenna height above the surface, and the target depth.

An investigation [35] into the use of bi-static radar signatures using experimental measurements showed that the separation between the antenna elements has a significant impact on the expected target return signal. From this it can be seen that the optimal separation may lie somewhere between 5 cm and 10 cm. In this experiment the antenna separation and target type were varied, while target depth, antenna height and soil type remained fixed.

In [36], an evaluation of GPR antennas is performed over real sand and soils. Here it was found that the optimal height for bowtie antennas was 4 cm, although this was the lowest value evaluated. Simulations of resitively loaded bowtie antennas were performed with a 3 cm antenna height. Here an antenna separation of 165 mm was chosen to reduce the direct coupling below 20 dB. However, these were designed with a significantly lower centre frequency (350 MHz) than those investigated in this paper (2 GHz), so that may not apply to this situation.

A study of the configuration of bi-static resistively-loaded dipole antennas at two frequencies, 300 MHz and 800 MHz, is presented in [37]. Here the antennas are evaluated both at end-to-end and side-by-side. The antenna height is evaluated at 0 cm, 5 cm and 10 cm, while the antenna separation is fixed. The authors conclude that the antennas should be kept as close as possible to the soil surface to ensure maximum coupling into the soil.

Related research on using air-launched bi-static GPR, using TEM Horn antennas, is presented in [38]. Another approach to solving some of the issues of shallow object detection is the introduction of a third antenna as shown in [39], which can reduce or eliminate the issues of direct coupling and ground reflection.

Work has been done in the past to create accurate models of bi-static GPR antenna systems and to match these to experimental measurements. This includes, for example, modelling of commercial GPR antennas inside gprMax [28,40]. In these experiments good agreement was reached between the simulations and experimental results. However, since the goal was to derive a model of commercial antenna systems, the antenna geometry was not varied.

The effects of the coupling between the antennas and the medium can also be modelled outside of finitedifference time-domain (FDTD) solvers such as gprMax, for example by using a set of point sources or infinitesimal dipoles to model a commercial bowtie antenna system [41] or Vivaldi antennas [42]; employing the Green’s functions to model near-field GPR data over planar layered media. A comparison between this approach and gprMax modelling can be found in [43].

Other research has focussed on modelling GPR antennas near lossy di-electrics and comparing the results to experimental measurements [44], and this work was extended to homogeneous and lossy heterogeneous soils in simulations [45]. Simulations on the detection of near-surface objects, in this case shallow buried land-mines, using realistic soil and soil surface models, were introduced in [46]. However only a single antenna configuration was investigated in this research.

There has also been considerable research to incorporate soil properties into GPR analysis and simulations. Stadler and Igel [47] studied a method using guided boreholes to measure the permittivity of the soil. Loewer and Igel [48] introduced a time domain reflectometry (TDR) method for an in situ assessment of the electrical properties of different soils, and then by implementing these findings into a soil simulation using gprMax software and a multi-pole Debye model [49].

This paper investigates the effects of the input impedance and geometrical configuration of bowtie antennas on the reflected signal strength from a near-surface object. The parameters investigated are the antenna separation and antenna height above the soil.

This paper is organised as follows. The first section introduces the set-ups of the simulation and measurement. The simulation and measurement results are then provided, including discussions of the optimisation of antenna input impedance, antenna performance testing, antenna height above the soil and antenna separation. Finally, the results are discussed, with suggestions for further research.

## 2. Methodology

The simulation software adopted in the experiment is gprMax [50], an open source electromagnetic simulation software using the FDTD method. The key parameters that were used in the simulations are shown in Table 1.

A Copper Mountain S5065 vector network analyser (VNA) was used to perform the measurements using step frequency continuous wave (SFCW) technology. Key parameters that were used in the measurements are shown in Table 2.

### 2.1. Individual Antennas

**Figure 1:**
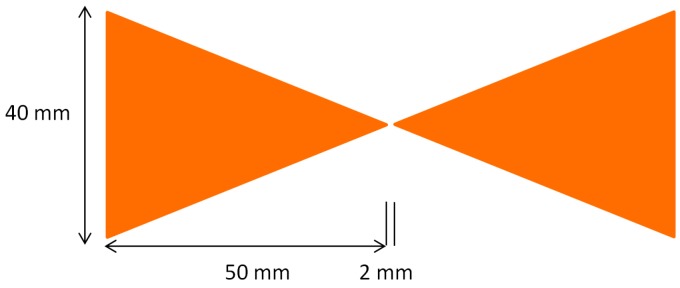
Dimensions of the solid bowtie antenna.

The bowtie antenna was chosen to be the antenna under examination in this paper, as discussed in the introduction. Like most GPR systems, the paper uses the bi-static antenna configuration. The antenna dimensions, for both simulation and measurement, are shown in Figure 1.

In the simulation, the antenna is represented by a sheet of perfect electrical conductor (PEC), using the surface of the appropriate voxels. The antennas were fed by a transmission line that can change the impedance in the simulation. In the measurement, the material of the bowtie antenna was copper, and the thickness of the copper on the PCB layer was 35 µm. The antennas were fed by a balun device that can transform the input impedance. The antenna size was chosen for a working frequency in the low GHz range, taking into consideration the antenna penetrating depth and resolution.

### 2.2. Object Description

**Figure 2:**
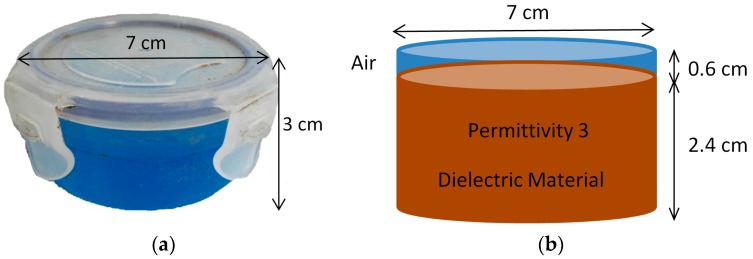
Object in the experiments: (**a**) Mine surrogate; (**b**) Object simulation.

The object in the experiments is a mine surrogate with a 7 cm diameter and 3 cm height, as shown in Figure 2. The surrogate is constructed from a polypropylene plastic tub partly filled to within 6 mm of the lid with a silicone material, with a relative permittivity of 3. In the simulation, the object is simulated as a plastic cylinder with an air layer above.

### 2.3. Soil Simulation

Previous work [51] simulated the soil as a homogenous loss-less soil with a relative permittivity of 20. This paper extended that work to cover both loss-less and lossy soil with two relative permittivities (8 and 20). A Debye model was used to simulate lossy soil [48], and the parameters of this model are shown in Table 3.

### 2.4. Face to Face Antenna Configuration

**Figure 3:**
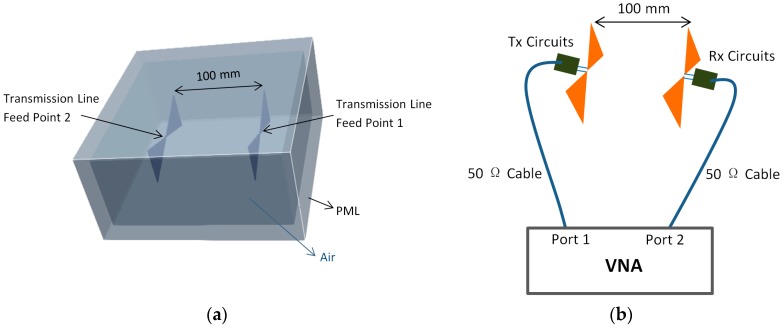
Setups for face-to-face experiments: (**a**) Simulation setup; (**b**) Measurement setup.

This simulation consists of two antennas facing each other at 100 mm distance in air, of which one is a transmitter, and the other is a receiver. This distance is representative of the typical depth for a near-surface object. In the simulation, a perfect matching layer (PML) was placed around the boundaries to absorb the electromagnetic waves. The simulation setup is shown in Figure 3a.

In the measurement, an antenna was connected to a 50 Ω transmission cable via a balun device that can transform the unbalanced signal in the cable to the balanced signal in the antenna and can change the input impedance of the antenna from 50 Ω to the desired value. Radar absorbing material (RAM) was placed around the antennas to reduce the interference, such as unwanted reflection from the background. The measurement setup is shown in Figure 3b.

### 2.5. Side by Side Antenna Configuration

**Figure 4:**
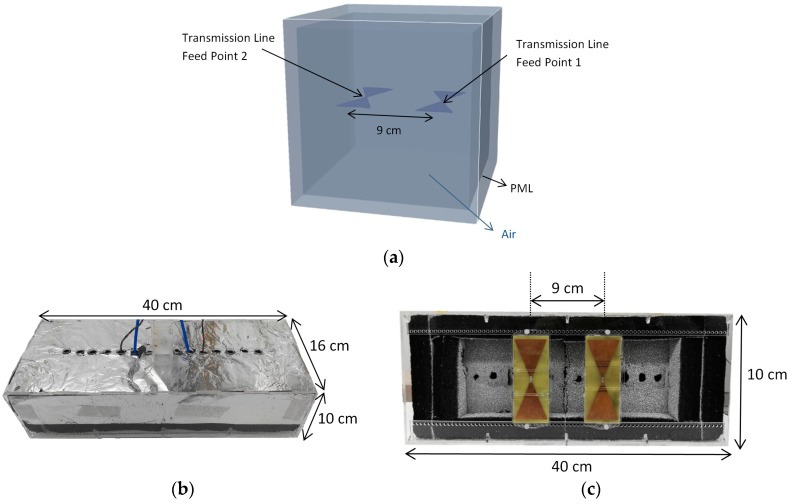
Setups for side-by-side experiments: (**a**) Simulation setup; (**b**) Radar housing top view; (**c**) Radar housing bottom view.

The antennas were also arranged in a side-by-side configuration in the air, as shown in Figure 4. The side-by-side orientation is the setup used when scanning the GPR in the field. For the measurement, the antennas were put into a radar housing filled with absorbing materials to prevent interference and unwanted reflections from above the ground. The radar housing was designed such that the separation between the bowtie antennas could be changed. In the example in Figure 4, this is shown with the nominal value of 9 cm.

**Figure 5:**
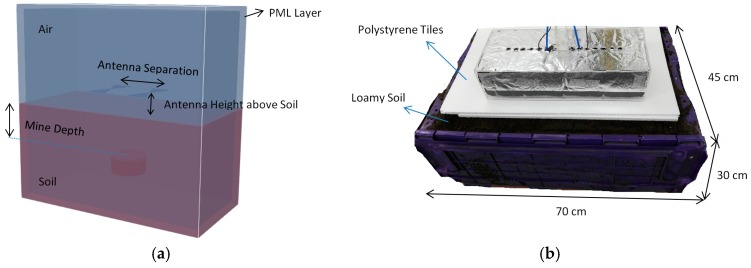
Side-by-side antennas above soil: (**a**) Simulation setup; (**b**) Measurement setup.

After the air test, the radar housing was placed above the soil. In the simulation (Figure 5a), side-by-side antennas were placed above the dielectric soil with or without lossy properties. A PML layer was used to absorb the electromagnetic waves on the external boundary of the air volume.

In the measurement (Figure 5b), the radar housing was placed above a tank filled with loamy soil. Low-density polystyrene tiles were placed between the radar housing and the soil surface to change the antenna height above the soil. Polystyrene tiles are transparent to the radio waves [52].

In the experiments above the soil, the parameters of the antenna were changed as listed in Table 4. Two-dimensional (2D) datasets were created by changing the antenna height above the soil and object depth with an antenna separation of 9 cm, and changing the antenna separation and object depth with an antenna height above soil at 3 cm. The soil surface was flat for all experiments and the given measurement of object depth relates to the depth of soil surface to the top of the object.

### 2.6. Data Processing

In the experiments, time-domain data was obtained by inverse fast Fourier transform (IFFT), applying zero padding and a Hamming window function to the complex frequency-domain data to calculate the time-domain signals.

To obtain the reflected signal purely from the object, two tests were performed for each simulation and measurement; the two tests were identical except one had the object in the soil while the other had no object. The difference between the two A-scan results was calculated, and this represents the reflected signal from the buried object.

The Hilbert transform was then applied to this difference signal to get the envelope of the reflected signal from the object. The maximum amplitude of the Hilbert transform was used in subsequent graphs, in which it represents the amount of signal reflected from the object.

When implementing the background subtraction, the simulation can calculate the true object signal, whereas it is not possible to subtract the true background clutter signal from the measurements and this process can only be approximated. In this paper, for deeper objects (>10 cm) the antenna cross-talk and ground bounce signals could be removed directly using a gating function since it was separated in time from the object reflection. For shallow objects (<10 cm), although some background clutter may have still been present in the calculated signal, it was small compared to the object reflection and thus can be ignored.

## 3. Results and Discussion

### 3.1. Antenna Input Impedance Simulation

The antenna input impedance was investigated using the face-to-face antenna configuration as described in the Section 2.4.

**Figure 6:**
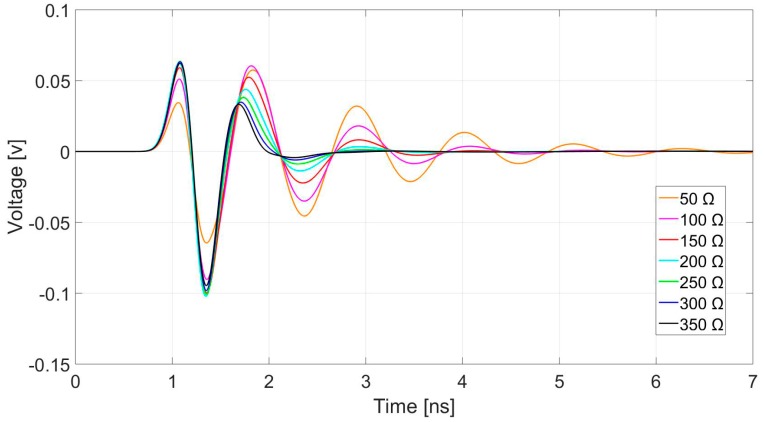
The effect of Antenna input impedance on late-time ringing.

In the simulation, the cable and balun structure was approximated by a transmission line with a given impedance. The characteristic impedance of the transmission line wave varied from 50 Ω to 350 Ω. The time-domain received signal (Figure 6) demonstrates that, in the air, the higher the input impedance, the higher the damping of the antenna, and hence the more compact the pulse shapes. For a GPR system, a short duration pulse can reduce the self-cluttering effect of the antenna and improve the distance resolution.

### 3.2. Comparison of Simulation and Experiment Waveforms

An impedance matching active-balun approach was used for the measurements. Due to PCB design considerations, the highest output impedance was found to be 250 Ω. The following results in the paper use this value for the characteristic line impedance for the simulation and the measurements.

**Figure 7:**
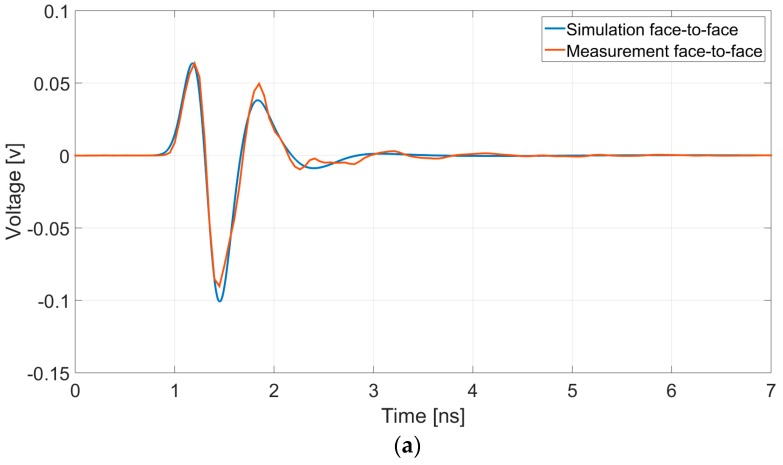

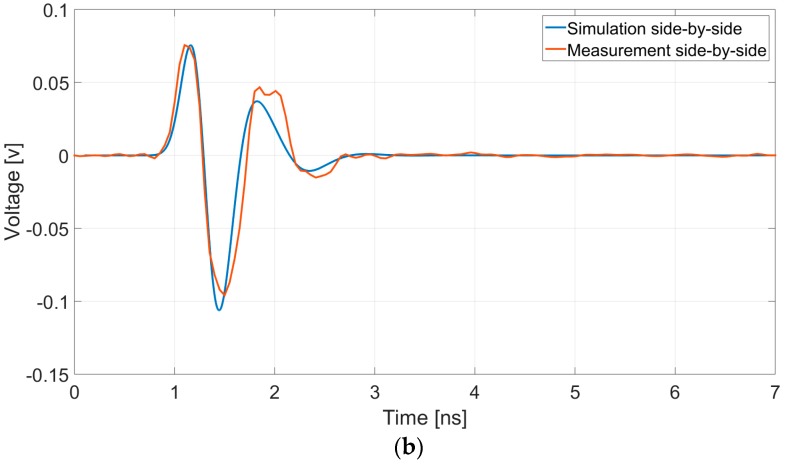
Comparison of simulation and measurement results for air testing: (**a**) Face-to-face configuration; (**b**) side-by-side configuration.

Figure 7 compares the results between the simulation and the measurements for the antennas in the air, in both the face-to-face configuration (Figure 7a), with an antenna separation of 10 cm, and the side-by-side configuration (Figure 7b) with a separation of 9 cm. The well-matched air testing results established the basis for further testing above the soil.

### 3.3. Antenna Height above the Soil

**Figure 8:**
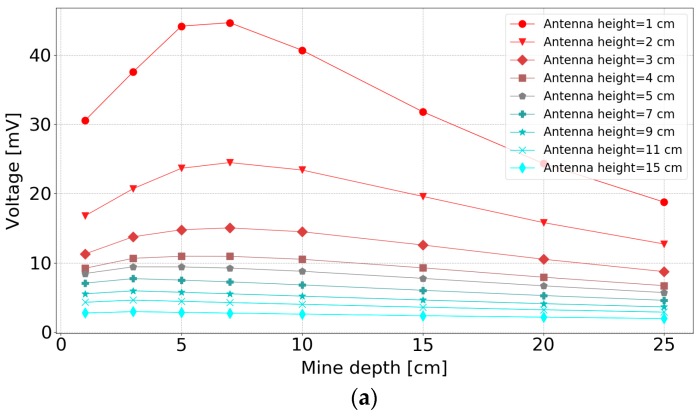

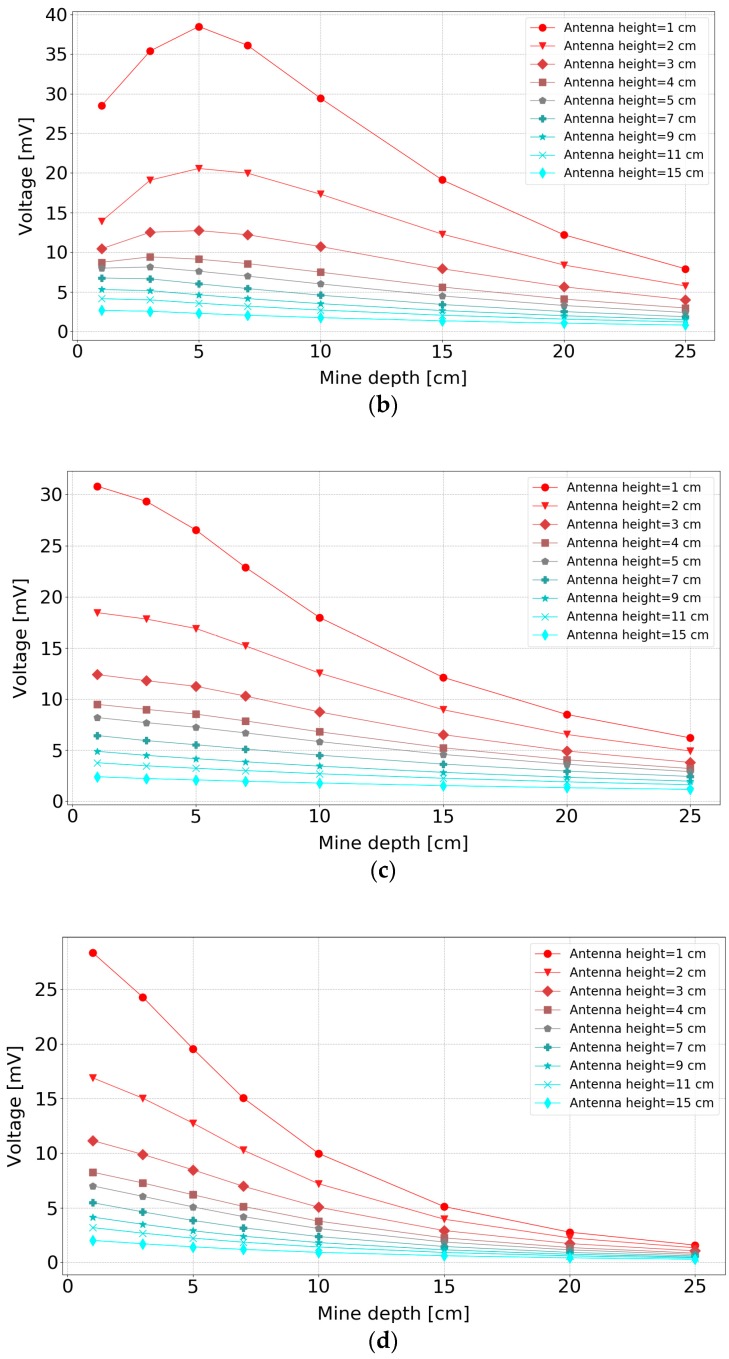

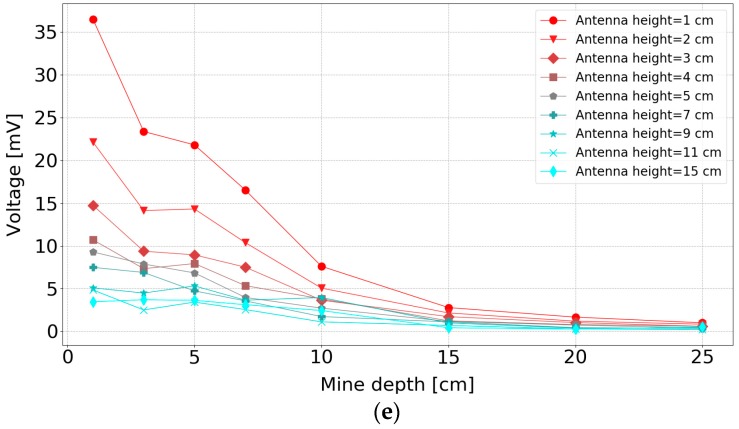
Antenna height above soil for a 9 cm antenna separation: (**a**) Lossless soil with a relative permittivity of 20 (simulation); (**b**) lossy soil with a relative permittivity of 20 (simulation); (**c**) loss-less soil with a relative permittivity of 8 (simulation); (**d**) lossy soil with a relative permittivity of 8 (simulation); (**e**) loamy soil (measurement).

This paper compares simulation results of antenna heights above the soil with measurement results above a loamy soil. The experiments varied both the antenna height above the soil and the object depth. The side-by-side antenna configuration was used for simulations and measurements. The results of these simulations are shown in Figure 8 for an antenna separation of 9 cm. One experiment dataset was measured above lossy loamy soil to verify the behaviour of the simulation (Figure 8e). The magnitude of voltage in Figure 8 represents the maximum absolute value of the Hilbert transform of the reflected signal from the buried object as explained in Section 2.6.

All the plots in Figure 8 show that the target signal reduces as the height of the antennas is increased. Naturally, large variations in signal response with the height above the ground are unwanted, which suggests that an operating height between 2 and 4 cm may be a good compromise between signal amplitude and stability of the height response.

Comparing Figure 8a and 8b shows that the lossy soil with a relative permittivity of 20 will reduce the mine reflected signal, especially for deep mines. A similar effect is seen comparing Figure 8c and 8d for the case when the ground relative permittivity is 8. The measured results in Figure 8e compare favourably with their simulated equivalent in Figure 8d.

**Figure 9:**
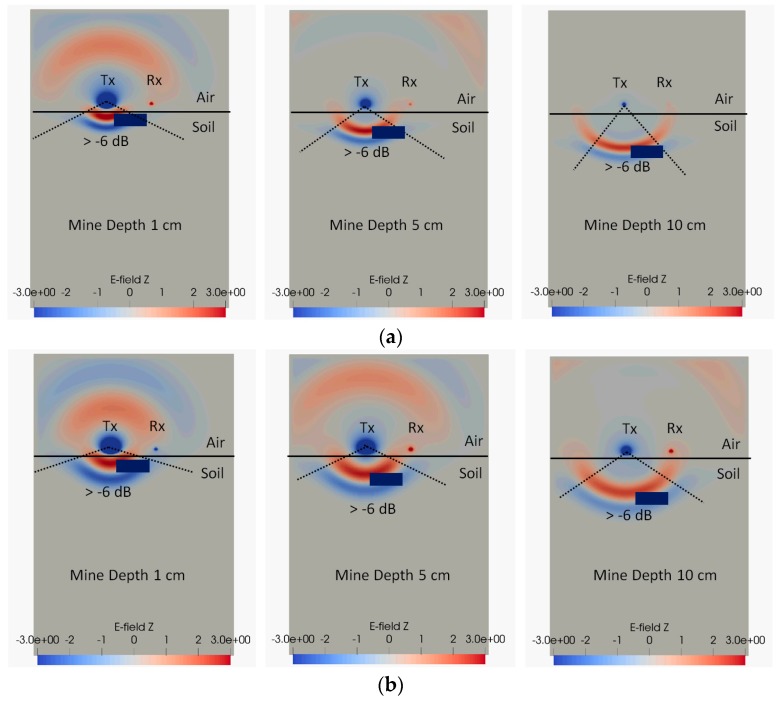
Radiating signal at different depths in lossy soil: (**a**) With a relative permittivity of 20; (**b**) with a relative permittivity of 8.

Figure 8 also demonstrates that, for an antenna separation of 9 cm, the shape of the radar curves is a function of soil permittivity. For a soil relative permittivity of 20 (both lossy and loss-less), the curves have a peak and the 5 cm deep object has the strongest response, while for the relative permittivity of 8 soil, no peak is shown and shallow objects give the strongest reflected signals. The reason for this difference is that as the soil permittivity increases, less energy is coupled into the ground at shallow angles close to the ground surface and consequently the response for shallow objects reduces [45]. To illustrate this phenomenon, simulation snapshots of the radiation pattern are shown in Figure 9. These figures demonstrate that for a soil relative permittivity of 20, the radiating beam has a narrow radiation pattern and the majority of radiated energy can “miss” the target as it travels in a more vertically downwards path. The situation is different for soil with a relative permittivity of 8, where the electromagnetic wave within the soil is more evenly spread with angle. The −6 dB angles are shown on the figures to emphasise this effect. Consequently, shallow targets in high permittivity soil will receive less radiation, resulting in the reduction of the radar signal shown for objects at depths below 5 cm in Figure 8a,b.

### 3.4. Antenna Separation

Another geometrical configuration that was investigated is the antenna separation. In this investigation, the antenna height was kept constant at 3 cm above the soil in the simulations and the measurements. The experiments varied the antenna separation and the object depth.

**Figure 10:**
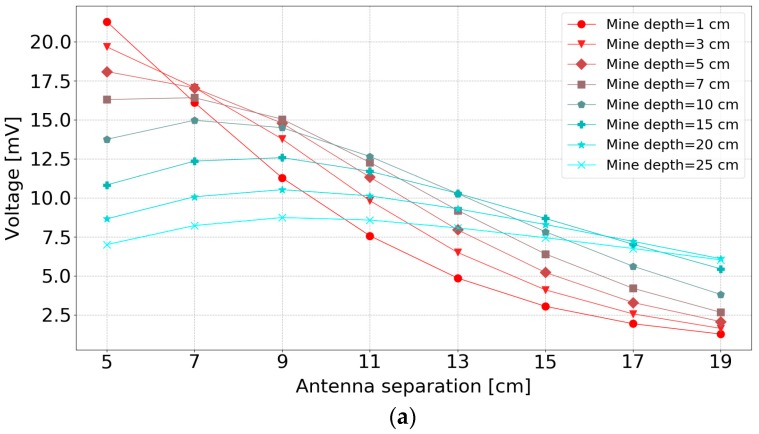

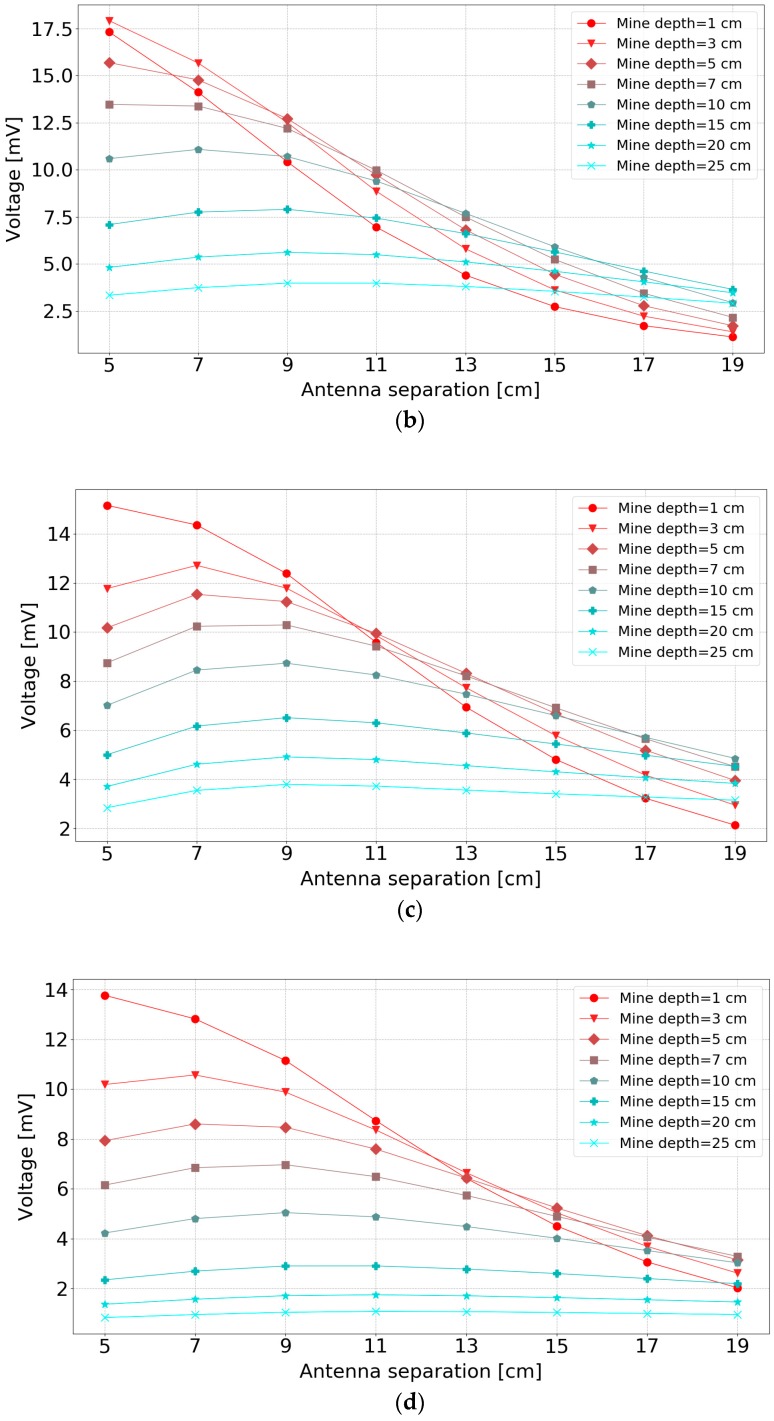

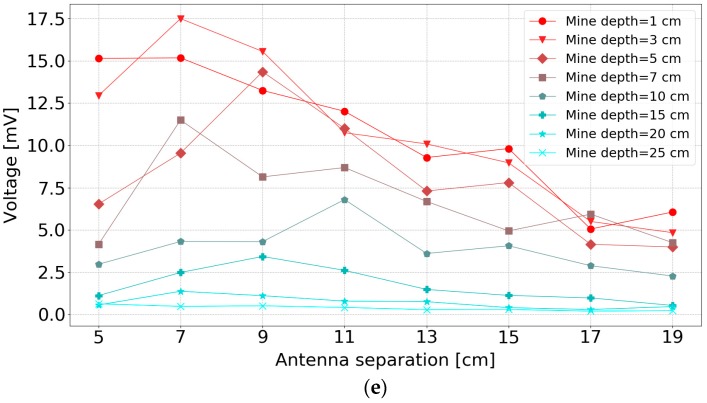
Results of antenna separation at a 3 cm lift-off height: (**a**) Lossless soil with a relative permittivity of 20 (simulation); (**b**) Lossy soil with a relative permittivity of 20 (simulation); (**c**) Lossless soil with a relative permittivity of 8 (simulation); (**d**) Lossy soil with a relative permittivity of 8 (simulation); (**e**) Loamy soil (measurement).

Figure 10a–d show antenna separation results above different soil simulations, and Figure 10e shows the measurements results above a loamy soil. The magnitude of voltage in Figure 10 represents the maximum absolute value of the Hilbert transform of the reflected signal from the buried object as explained in Section 2.6.

The general trend is that the received signal decreases with increasing antenna separation. However, the maximum reflection amplitude occurs for an antenna separation of between, approximately, 7 cm to 9 cm.

**Figure 11:**
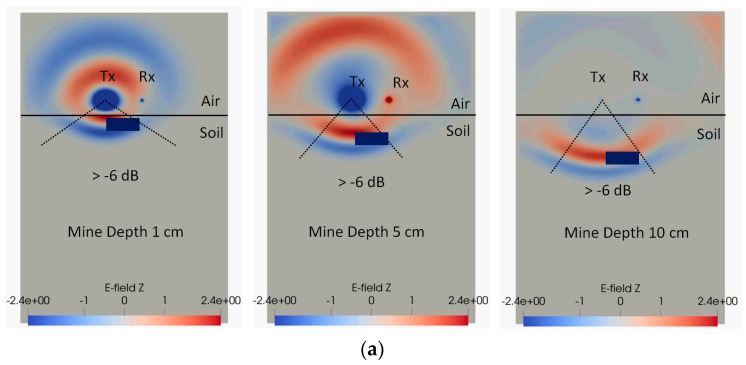

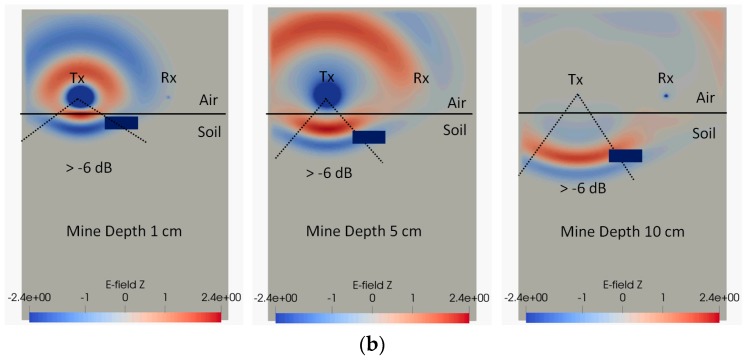
Radiating signal at different depths in loss-less soil (relative permittivity of 8) with 3 cm antenna lift-off height: (**a**) Antenna separation 7 cm; (**b**) Antenna separation 17 cm.

For a smaller separation (<7 cm), the received voltage peak decreases with reflecting object depth. However, this situation starts to reverse for large bi-static antenna separation, especially with higher permittivity soils. This causes intersections of lines for shallow and deep mines in Figure 10. The reason for this phenomenon is explained as shown in Figure 11. With the increase of the antenna separation, the amount of wave energy hitting the object and then reaching the antenna is decreased when the object is nearer the surface. This effect is exacerbated by the angular dependence of the receiver sensitivity. The effect tends to become more obvious when the antenna separation is greater than the mine depth, and for deeper mines, the effect of antenna separation becomes smaller. For lossy soils, the amplitude of the reflecting objects is decreased for deep mines, due to the partial absorption of the wave by the soil, so in this case, the intersection effect is reduced.

Therefore, considering the stability for all mine depths and to get a relatively bigger response, the antenna separation was chosen to be between 7 cm to 9 cm to get the large reflected signal for both deep and shallow mines.

In the measurement dataset, Figure 10e, the antenna sensor head needed to be reassembled for each antenna separation, which had the consequence of less repeatable measurements. Although these measurements appear to be quite noisy, the trend of the measurements follows the trends of the simulation of lossy soil with a relative permittivity of 8.

The lossy soil properties reduce the signals from the deep mine significantly. However, higher soil permittivity can increase the reflected signal for deep mines, due to a larger difference between the permittivity of soil and mine.

### 3.5. In-Field Testing

**Figure 12:**
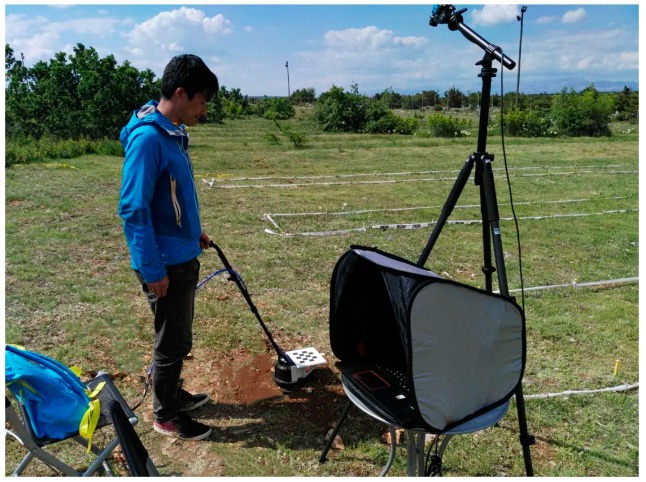
Field test in Croatia.

After deciding on the antenna geometry, field tests were performed at the Benkovac test site in Croatia (Figure 12) during September 2018 to verify the design and confirm the efficacy of the suggested geometry in practical conditions.

The test site, established in 2000, is owned by HCR-CTRO and it consists of blind test lanes and training lanes. A representative example of the response of the GPR system from a training lane that contained homogenous soil is shown here. In this example, the target object was a PMA3 anti-personnel landmine. The position of the radar head was measured with a camera position measuring system that has 1 cm resolution to produce C-scan datasets.

The antenna height above the ground was chosen to be 3 cm as explained in Section 3.3, while the antenna separation was chosen to be 7 cm, due to the design results detailed in Section 3.4 above.

The GPR head also contained a carefully designed septum between the transmitter and the receiver to reduce the direct cross-talk between the transmitter and receiver, and to improve the ratio of the object signal and the cross-talk signal.

To transform the frequency-domain measurements to the time-domain, an inverse Fourier transform was used, after applying a Hamming window function and zero-padding the data. The direct coupling between the antennas and the reflection from the ground were removed using a background removal algorithm, which subtracts the ensemble mean of all A scans from each A scan. Finally a time-varying gain was applied to the signal to account for the spreading losses. Table 5 shows the acquisition parameters used during the field trial. Note that the intermediate frequency (I.F.) bandwidth is increased to reach a higher sweep speed with the VNA.

**Figure 13:**
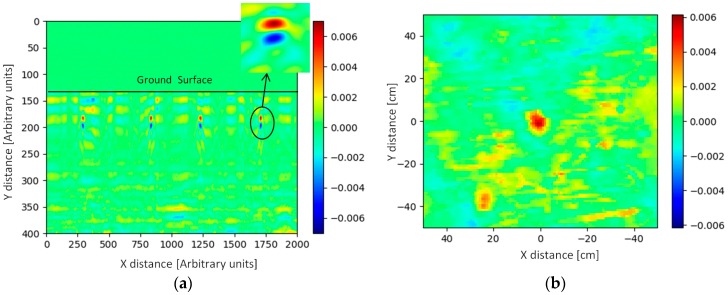
Results for PMA3 buried at 5 cm (measurement): (**a**) B scan; (**b**) C scan.

Figure 13a shows the B scan results for the PMA3 target buried at a depth of 5 cm in the soil; in this case, the detector has been scanned four times over the object. Figure 13b shows the C scan results making use of the concurrent positional data from the camera system. Here, two mines buried at a 5 cm depth can be seen; a PMA2 mine on the left bottom corner and PMA3 in the middle. The PMA2 is a physically smaller device and hence gives a smaller GPR response. The colour-bar in Figure 13 represents the arbitrary units of the signal amplitude after signal processing.

## 4. Conclusions

In this paper, the effect of the geometrical configuration of bi-static bowtie antennas on reflected signal strength from near-surface objects was investigated, both in simulations and with measurements. Specifically, the effects of antenna separation and antenna height above the soil were explored, for multiple soil types and a range of depths for the near-surface object. A small side study was completed to find the optimal input impedance for the antennas, with the aim of reducing the late-time ringing from the antennas.

A small side study was also conducted to find the optimal input impedance for the antennas, with the aim of reducing the late-time ringing from the antennas. It was found that the late-time ringing reduces with higher input impedance. For this paper, the input impedance was chosen to be 250 Ω, due to practical limitations on the highest output impedance achievable on the antenna feed. The issue of the optimal input impedance would merit further research, as it is an important factor in determining the radiating pulse shape.

Based on the experiments performed during this study it can be seen that an antenna height of 2 cm to 4 cm above the soil, and an antenna separation of 7 cm to 9 cm, are a good choice for near-surface object detection. The results indicate that the geometry of the bi-static antennas is important to the performance of GPR systems.

It was also shown that the relationship between the reflected signal, and the antenna height and mine depth, varies significantly with the soil properties. For high permittivity soils, the relationship between peak reflected voltage and mine depth is non-monotonic. This is likely caused by a lensing effect at the air-ground interface, due to higher angles of refraction, leading to narrow beam angles in the soil at higher permittivity.

The measured results for this experiment showed good agreement with the trends found from the simulations. The results were used to guide the design of a GPR system for near surface target detection, and the field results confirmed that the choice of the antenna geometry was able to detect mines in field conditions. The investigation performed in this paper is intended for the development of a GPR system using solid bowtie antennas for near-surface object detection. Although the results are aimed at proximal GPR systems, the results may also be useful in the design of other GPR systems.

The soil surface was flat in the laboratory-based measurement and the simulations. In the field tests, the undulating soil surface impacts the antenna height above the soil and therefore changes the performance of the GPR system. Soil surface undulation makes background subtraction more difficult. GPR system designers should be aware of this problem so that they can account for it in their processing algorithms, and carefully choose the optimal antenna height above the soil.

## Figures and Tables

**Table 1:** Summary of the key simulation parameters used.

gprMax Parameters	Value
Frequency range	20 MHz to 6 GHz
Time domain range	20 ns
Input signal format	Gaussian signal
Input signal amplitude	1 V
Input signal central frequency	1.5 GHz
Domain dimension	40 cm × 60 cm × 20 cm
Cell size	2 mm
Perfect matching layer (PML) thickness	10 cells

**Table 2:** Summary of the key vector network analyser (VNA) parameters used.

VNA Parameters	Value
Number of steps	201
Frequency step size	32.3 MHz
Start frequency	32.3 MHz
Stop frequency	6500 MHz
I.F. bandwidth ^1^	1 kHz

^1^ I.F. bandwidth is short for intermediate frequency bandwidth.

**Table 3:** Debye model parameters for the loamy soil simulation.

Parameters	Value
ɛ∞	3.32
Δε_1_	4.35
τ_1_ (ps)	5.90
Δε_2_	53.34
τ_2_ (υs)	428.7
σ_dc_ (S/m)	0.012

Where: ɛ∞ is the relative permittivity at infinite frequency; Δε_1_ is the difference between the zero-frequency relative permittivity and the relative permittivity at infinite frequency for the first Debye pole; τ_1_ is the relaxation time for the first Debye pole; Δε_2_ is the difference between the zero-frequency relative permittivity and the relative permittivity at infinite frequency for the second Debye pole; τ_2_ is the relaxation time for the second Debye pole; σ_dc_ is the dc conductivity of the soil.

**Table 4:** Variables for geometrical configuration experiments.

Variables	Nominal Value (cm)	Variable Range (cm)
Antenna separation	9	[5, 7, 9, 11, 13, 15, 17, 19]
Antenna height above soil	3	[1, 2, 3, 4, 5, 7, 9, 11, 15]
Object depth	10	[1, 3, 5, 7, 10, 15, 20, 25]

**Table 5:** Summary of the key VNA parameters used in field testing.

VNA Parameters	Value
Number of steps	200
Frequency step size	30 MHz
Start frequency	30 MHz
Stop frequency	6000 MHz
I.F. bandwidth ^1^	100 kHz

^1^ I.F. bandwidth is short for intermediate frequency bandwidth.
